# Course of Disease and Clinical Management of Patients with Poorly Differentiated Thyroid Carcinoma

**DOI:** 10.3390/cancers13215309

**Published:** 2021-10-22

**Authors:** Freba Grawe, Atika Cahya, Matthias P. Fabritius, Leonie Beyer, Vera Wenter, Johannes Ruebenthaler, Thomas Geyer, Caroline Burgard, Peter Bartenstein, Harun Ilhan, Christine Spitzweg, Andrei Todica

**Affiliations:** 1 Department of Nuclear Medicine, University Hospital, LMU Munich, 81377 Munich, Germany; Freba.Ahmaddy@med.uni-muenchen.de (F.G.); atikacahya@gmail.com (A.C.); Leonie.Beyer@med.uni-muenchen.de (L.B.); Vera.Wenter@med.uni-muenchen.de (V.W.); Caroline.Burgard@med.uni-muenchen.de (C.B.); Peter.Bartenstein@med.uni-muenchen.de (P.B.); Harun.Ilhan@med.uni-muenchen.de (H.I.); 2 Department of Radiology, University Hospital, LMU Munich, 81377 Munich, Germany; Matthias.Fabritius@med.uni-muenchen.de (M.P.F.); Johannes.Ruebenthaler@med.uni-muenchen.de (J.R.); Thomas.Geyer@med.uni-muenchen.de (T.G.); 3 Comprehensive Cancer Center (CCC LMU) and Interdisciplinary Center for Thyroid Carcinoma (ISKUM), University Hospital, LMU Munich, 81377 Munich, Germany; Christine.Spitzweg@med.uni-muenchen.de; 4 Department of Internal Medicine IV, University Hospital, LMU Munich, 81377 Munich, Germany

**Keywords:** poorly differentiated thyroid cancer, ^18^F-FDG-PET/CT, radioiodine therapy, PDTC, risk stratification

## Abstract

**Simple Summary:**

Poorly differentiated thyroid carcinoma (PDTC) represents a rare but aggressive variant of thyroid carcinoma and contributes to a significant proportion of thyroid carcinoma-associated deaths. Studies on PDTC are rare; therefore, we aim to assess the clinical course of these patients, evaluate the prognostic value of response to initial radioiodine therapy and identify risk factors for poor prognosis to optimize the clinical management of patients with PDCT.

**Abstract:**

Background: In patients with poorly differentiated thyroid carcinoma, the clinical course and prognostic value of response to initial radioiodine therapy is evaluated. Methods: In 47 patients, clinical and imaging features were analyzed. Patients were stratified in no (NED), biochemical (B-ED) and structural evidence of disease (S-ED) assessed at the first diagnostic control and its impact on survival was evaluated. Further, possible risk factors for a shorter disease-specific survival rate (DSS) were analyzed. Results: In total, 17/47 patients consisted of NED, 10/47 were B-ED and 20/47 S-ED patients. At the last follow-up, 18/47 patients were NED, 2/47 patients B-ED and 27/47 patients S-ED. The median survival time was only reached for the S-ED group (median 3.9 years, 95%CI 2.8–5.1 years) and was not reached in the B-ED and NED groups. Metastases were diagnosed by a ^18^F-FDG-PET/CT scan in all cases and a multivariate analysis showed that the PET-positivity of metastases was the only significant predictor of DSS (*p* = 0.036). Conclusion: The response to initial surgery and radioiodine therapy in PDTC patients can achieve an excellent outcome and a further follow-up should be refined based on findings at the first diagnostic control. However, patients with an incomplete response and metastatic patients who become mostly radioiodine refractory show a significantly shorter survival, which makes accurate staging by ^18^F-FDG-PET/CT imaging crucial.

## 1. Introduction

Poorly differentiated thyroid carcinoma (PDTC) represents a rare (2–15%) but aggressive variant of thyroid carcinoma and contributes to a significant proportion of thyroid carcinoma-associated deaths [1]. In 2004, the World Health Organization (WHO) classification [2], which was updated in 2017 [3] and the Turin proposal in 2006 [4], defined PDTC as a combination of specific features of cell structure (solid, trabecular or insular growth pattern and nuclear changes) and features of malignancy (vascular invasion and/or invasive growth) [5]. PDTC shows not only the morphological but also biological characteristics that lie between the well-differentiated (DTC) and undifferentiated anaplastic thyroid carcinoma (ATC), which results in a corresponding clinical course and prognosis that can vary between fairly mild courses up to very aggressive behavior [6]. The 5-year disease-specific survival rate (DSS) in patients with PTDC (51%) is between that of patients with DTC (91%) and with ATC (0%) [7]. In DTC, due to the preserved differentiation of tumor cells and, therefore, their ability to accumulate I-131, the established therapeutic approach includes a primary surgical tumor resection followed by a risk-adapted application of radioactive iodine (RAI) therapy to eliminate residual thyroid tissue and undetectable microscopic tumor lesions [8]. ATC remains a devastating diagnosis with very limited therapeutic options with a glimmer of hope with new approaches based on a genomic interrogation of tumors and incorporating biologically targeted agents and immunotherapeutics in multimodality therapy protocols [9]. In PDTC, lesions often lose their ability to concentrate I-131, which can result in a decreased RAI avidity and, therefore, only limited effectiveness of RAI therapy. However, with the loss of RAI avidity, PDTC develops 2-deoxy-2-fluoro-D-glucose (^18^F-FDG)-positivity, detected by positron-emission-tomography (PET) [10], which is reported to be associated with more aggressive tumor behavior and a worse outcome [11]. Previous studies have shown that the loco-regional status does not lead to death, but distant metastases do (mostly lung and bone), which makes accurate staging in PDTC patients crucial. Therefore, the aim of this study is to assess the clinical course of patients with this relatively rare subtype of thyroid cancer and to identify risk factors for poor prognosis. Furthermore, we evaluate the prognostic value of the response to initial RAI therapy and opted to find the best clinical management of PDTC patients.

## 2. Materials and Methods

### 2.1. Patient Characteristics

In this retrospective study, 1619 consecutive patients who underwent total thyroidectomy followed by RAI therapy at the Department of Nuclear Medicine (University Hospital, LMU Munich) between 2005 and 2019 were screened. Pathological reports from the referring hospitals were reviewed for PDTC. Patients were included if they had at least the first diagnostic control (i.e., evaluation of ablation success) at 6 to 9 months after initial RAI therapy. Further, in all patients, at least one ^18^F-FDG-PET/CT scan was performed in the time span between initial RAI therapy and first diagnostic control. The final study population consisted of 47 patients with pathologically confirmed PDTC. Epidemiological and clinical features (e.g., age, gender, tumor stage) of study patients were assessed. Tumor-node-metastasis (TNM) staging was based on the seventh edition of the American Joint Committee on Cancer (AJCC).

### 2.2. Treatment 

All patients underwent total thyroidectomy with additional lymphadenectomy in cases of preoperatively suspected and/or intraoperative proven lymph node metastases. Initial (adjuvant) RAI therapy was administered ranging from 3.7 to 7.4 GBq I-131 depending on the initial recurrence risk status [12]. Prior to RAI therapy, patients were stimulated with recombinant human thyroid-stimulating hormone (rhTSH, Thyrogen^®^, Sanofi Genzyme, Cambridge, MA, USA) i.m. on two consecutive days or underwent hormone withdrawal prior to RAI therapy to achieve TSH levels ≥ 30 µU/mL according to current German and European guideline recommendations [13].

### 2.3. Follow-Up and Outcome Analysis

First diagnostic control (evaluation of ablation success) was assessed at 6 to 9 months after initial RAI therapy, including neck ultrasound, determination of stimulated Tg-level and diagnostic I-131 whole body scintigraphy (WBS). WBS was performed approximately 72 h after application of 370 MBq I-131 in hypothyroidism or after administration of rhTSH i.m. on two consecutive days. In case of any pathological finding in the WBS, an additional single-photon-emission computed-tomography/low-dose computer-tomography (SPECT/CT) of the relevant region was performed (most often neck and thorax). Furthermore, in all patients with PDTC, at least one ^18^F-FDG-PET/CT was performed between initial RAI therapy and first outcome evaluation. Further follow-up examinations included regular laboratory and ultrasound check-ups every 3 months in the first year after RAI therapy, every 6 months in the second year and annually thereafter. In case of suspicious findings during follow-up, patients were monitored more closely.

Radioiodine refractory patients were defined by the presence of at least one metastatic site without any uptake of radioiodine, disease progression during the first year after a radioiodine treatment course or the persistence of disease after the administration of a cumulative activity of 22 GBq I-131. Patients were classified as no evidence of disease (NED) at first outcome evaluation as a combination of stimulated Tg < 0.5 ng/mL and no relevant uptake in the I-131 whole body scan. Neck ultrasonography was performed in all patients but not further evaluated in this study. Persistent biochemical disease (B-ED) was defined as a continued elevated stimulated or non-stimulated Tg ≥ 0.5 ng/mL with no decrease under the detection limit after RAI therapy without structural correlate. Persistent structural disease (S-ED) included patients with elevated stimulated or non-stimulated Tg-level and tumor tissue detected by morphological imaging (WBS or ^18^F-FDG-PET/CT). Recurrence was assumed if Tg-level increased above the detection limit or if tumor lesions were detected by morphological imaging after complete response in the first follow-up examination. Disease-specific survival was defined as the time between thyroidectomy until disease-specific death.

### 2.4. Statistical Analysis

All continuous variables were expressed as mean ± standard deviation. Survival analysis was performed using Kaplan–Meier analysis for DSS. Log-rank test was used to compare survival rates between subgroups. Survival was displayed as mean (95%CI) if median survival was not reached. Quantitative survival data are given as median in years. Parameters that showed significant influence on DSS in the univariate analysis were included in the multivariate analysis. The multivariate regression model was applied to analyze prognostic factors associated with DSS. A *p*-value < 0.05 was considered statistically significant. All analyses were performed using SPSS computer software (SPSS Statistics 25, IBM, Armonk, NY, USA).

## 3. Results

### 3.1. Patient Characteristics

In this study, 47 patients (26 female) with PDTC were included. The mean age at treatment start was 57 ± 19 years, with 28/47 patients being 55 years or older. At the initial presentation, most patients had pT3-stage (34/47), N0/Nx-stage (33/47), were R0/Rx-resected (37/47) and had no extrathyroidal extension (ETE, 9/47) according to the 7th edition of the American Joint Committee on Cancer (AJCC)/TNM classification. Prior to the RAI treatment, 24 patients were stimulated with recombinant human TSH and 23 patients underwent hormone withdrawal to achieve TSH levels ≥ 30 µU/mL. RAI therapy was performed with an initial dose of 6.2 ± 1.9 GBq I-131. The stimulated mean Tg-level at initial RAI therapy was 1305 ng/mL (<0.05–4109 ng/mL). Ten patients initially presented with distant metastases: seven patients with pulmonary metastases, one with bone metastases and two with pulmonary and bone metastases. Radioiodine avidity was seen in 4/9 pulmonary metastases and 2/3 bone metastases. A ^18^F-FDG-PET/CT scan was performed in 24/47 patients in the time span between the initial RAI therapy and 3-month follow-up examination, showing a pathological ^18^F-FDG uptake in all patients with distant metastases at the primary presentation. Patient characteristics are summarized in Table 1 and TNM classification in Table 2.

### 3.2. First Diagnostic Control

**Figure 1:**
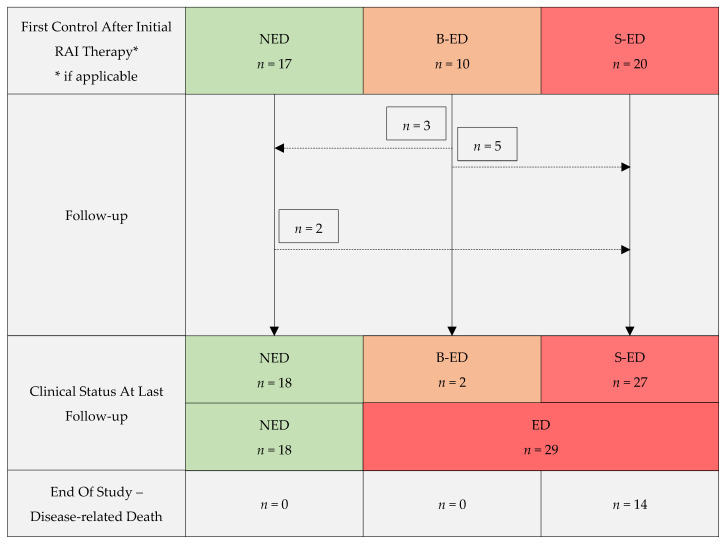
Risk stratification of patients with poorly differentiated thyroid carcinoma; ED, evidence of disease; N, no; B, biochemical; S, structural.

In the first diagnostic control after RAI therapy, three subgroups of patients could be identified—patients with NED highlighted in green, B-ED in orange and S-ED in red (see Figure 1). The NED-subgroup consisted of 17/47 patients. In 4/17 patients, I-131 uptake in the thyroid bed was diagnosed in the WBS without detectable Tg or a pathological ^18^F-FDG uptake in the PET/CT scan and, thus, corresponding to non-pathological, remnant thyroidal tissue. In these patients, an additional second course of RAI therapy was performed, and patients counted among the NED group until the last follow-up. Ten of forty-seven patients showed B-ED with a mean stimulated Tg of 13.1 ng/mL (0.8–85.7 ng/mL) without pathological findings in the WBS. In 2 of these 10 patients, I-131 uptake in the thyroid bed was detected without corresponding findings in the ^18^F-FDG-PET/CT scan, confirming non-pathological remnant thyroidal tissue. The S-ED group consisted of 20/47 patients with I-131 uptake in the WBS found in 12 patients (lung = 7, remnant thyroidal tissue = 4, bone = 3, liver = 1). The ^18^F-FDG-PET/CT scan at the first diagnostic control was performed in 16/20 patients and confirmed distant metastases in all patients. In addition, further distant metastases (lung = 3, lymph node = 3, pleura = 1) could be detected. In two of four patients with I-131 uptake in the thyroid bed, local disease could be confirmed in the ^18^F-FDG-PET/CT scan. In all remaining patients of the S-ED, as well as the B-ED and NED group, the ^18^F-FDG-PET/CT scan was performed either at the initial presentation (as reported in the patient characteristics section) or at the 3-month follow-up. The mean stimulated Tg in patients of this group was 1134.7 ng/mL (<0.05–10,063.0 ng/mL). Seven patients did not receive a first diagnostic control due to extended metastatic disease diagnosed by the WBS or ^18^F-FDG-PET/CT scan at initial RAI therapy. Two patients died before first the diagnostic control. In two patients, systemic therapy (tyrosine kinase inhibitor, TKI) was initiated immediately after initial RAI therapy.

### 3.3. Outcome during Follow-Up

In the NED group, 15/17 patients remained NED until the last follow-up. In two patients, structural recurrence, diagnosed by a ^18^F-FDG-PET/CT scan, occurred after 2.3 and 12.1 years, respectively. Despite the multimodal treatment, including further RAI therapy cycles, surgery, radiotherapy and systemic therapy, the outcome could not be improved during the follow-up and metastases continued to spread (lung = 2, pleura = 2, brain = 1, bone = 1 and liver = 1).

In the B-ED group, three patients showed no further Tg-levels above the detection rate after additional RAI therapy (*n* = 3), confirming Tg due to remnant thyroidal tissue. Until the last follow-up, two patients remained in the B-ED group after local radiotherapy and combined radiotherapy with additional RAI therapy, respectively. It should be emphasized that, in patients with pathologically confirmed PDTC and extended local disease (pT-stage ≥ 3), radiotherapy was performed frequently at the time of inclusion. The non-stimulated Tg at the last follow-up was 0.6 ng/mL in one patient and 94.6 ng/mL in the other patient. In five patients, structural disease occurred during the follow-up after a mean of 3.0 ± 2.0 years. One patient showed lymph node metastases, one showed bone metastases and three patients had more than one site of distant metastases, including one patient with a local recurrence, all detected by a ^18^F-FDG-PET/CT scan. After multimodal therapy, including further cycles of RAI therapy, only one patient showed iodine-avid bone metastases, whereas all other patients were radioiodine refractory. The mean non-stimulated Tg at the last follow-up was 2302.8 ng/mL (21.2–11,262.0 ng/mL).

All patients with detectable Tg-levels and structural disease (20/47) remained in the S-ED group until the last follow-up. The mean Tg at the last follow-up was 3539.5 ng/mL (<0.05–19,097.0 ng/mL). Metastases were diagnosed by a ^18^F-FDG-PET/CT scan in all cases. The leading metastatic site was the lung (*n* = 16), followed by lymph nodes (*n* = 12) and bones (*n* = 12). An additional local recurrence was found in 5/20 patients. Systemic therapy (e.g., TKIs Sorafenib and Lenvatinib) was performed in nine patients and was planned in one additional patient at the end of the study, seven patients were re-operated, seven patients received additional cycles of RAI therapy, six patients received radiotherapy and four patients were treated with combined RAI therapy and radiotherapy. One patient refused further therapy. The majority of patients treated with further RAI therapy cycles became radioiodine refractory during treatment (*n* = 10/11).

### 3.4. Survival Analysis

**Figure 2:**
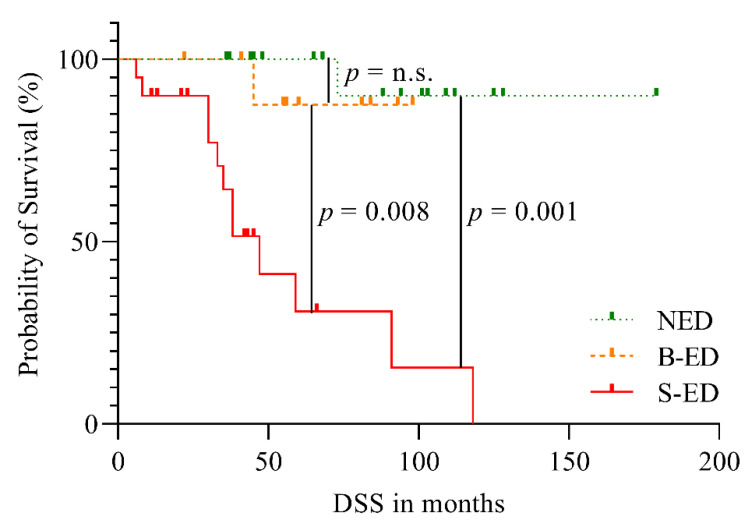
Kaplan–Meier estimate of DSS in the NED, B-ED and S-ED groups; DSS, disease-specific survival; ED, evidence of disease; N, no; B, biochemical; S, structural, n.s., not significant.

The mean follow-up time was 5.1 ± 3.1 years. At the end of the study, 16/17 patients of the initial NED group were alive. One patient who developed S-ED during the follow-up died of thyroid cancer. Of the initial B-ED group, 9/10 patients were alive at the final follow-up and one patient who developed S-ED died of thyroid cancer. Of the initial S-ED patients, 14/20 had died of thyroid cancer (see Figure 1). At the last follow-up, 18/47 patients presented with NED, 2/47 patients with B-ED and 27/47 with S-ED. Respiratory failure and brain hemorrhage were the most common causes of death. The median survival time was only reached for the S-ED group (median 3.9 years, 95%CI 2.8–5.1 years) and was not reached in the B-ED and NED group (see Figure 2). The S-ED group survival was significantly reduced compared to the NED group (*p* = 0.001) and the B-ED group (*p* = 0.008). Four patients showing disease progression were lost to follow-up.

### 3.5. Prognostic Factors Associated with DSS

To analyze possible risk factors for shorter DSS, we performed univariate and multivariate analyses. In univariate analyses of the baseline variables on the primary presentation, only age ≥ 55 years (*p* = 0.045) and at M1 stage (*p* = 0.001) were associated with a significantly shorter DSS, whereas gender, the presence of ETE, pT stage ≥ pT3, N1a/N1b stage and R1/R2 stage did not influence survival. Stimulated Tg ≥ 0.5 ng/mL and I-131 dose of ≥7400 MBq at initial RAI therapy also showed no statistically significant difference in survival. At the first control, after initial RAI therapy, stimulated Tg ≥ 0.5 ng/mL (*p* = 0.011) and PET-positivity of metastases (*p* = 0.012) were unfavorable prognostic factors for DSS. The RAI avidity of metastases had no significant effect on the survival. The multivariate analysis showed that the PET-positivity of metastases (*p* = 0.036) was the only significant predictor of DSS. All significant risk factors for DSS are presented in Table 3.

## 4. Discussion

PDTC comprises a small subset of thyroid carcinomas but contributes to a significant proportion of thyroid carcinoma-associated deaths. Due to the rarity of the disease, studies on PDTC patients are limited. The aim of our study was to investigate the clinical course of PDTC patients and possible prognostic factors for a poor outcome. We found that the response to the initial RAI therapy determined the further course of disease and DSS. Furthermore, we identified PET-positivity of distant metastases as a prognostic factor for poor outcome.

As part of the initial staging, patients with DTC could be stratified in three categories for their risk of recurrence according to the American Thyroid Association (ATA) guidelines—low, intermediate and high risk of recurrence [13]. Since the initial surgery and RAI therapy had a profound impact on the further clinical course of patients, but finding no mention in initial staging systems, we stratified PDTC patients in three risk groups (NED, B-ED and S-ED) assessed at the first diagnostic control in an attempt to investigate its impact on survival (DSS). Indeed, patients with a complete response after the initial therapy (NED group) showed the longest survival time, followed by patients with biochemical disease (B-ED group) and, last, patients with structural disease (S-ED group). Among the groups, S-ED patients showed significantly reduced DSS compared to B-ED and NED patients, whereas survival between the B-ED and NED group did not differ significantly. Tuttle et al. [14] already demonstrated the usefulness of dynamic risk stratification in patients with DTC; however, PDTC was not included in this study. At the end of the study, the majority of patients with NED in the first diagnostic control remained cured until the end of the study, which corresponded to around one third of study patients (18/47 patients). This finding was in line with a study by de la Fouchardière et al. [15], who also reported one third of cured patients of their study cohort after initial surgery and RAI therapy. Nevertheless, two of our study patients of the NED group at first diagnostic control developed structural disease and one of them died of their disease, which makes a periodic, lifelong follow-up crucial in PDTC patients.

In the B-ED group, a heterogonous course of the disease was observed. While half of the patients (5/10 patients) developed structural disease during the follow-up, only one of these patients died at the end of the study. In contrast, in the initial S-ED group, most patients died by the end of the study (14/20 patients). Three of ten patients in the B-ED group received a second RAI therapy or local radiation and were NED until the end of the study. Until the last follow-up, two patients remained in the B-ED group after local radiotherapy and combined radiotherapy with additional RAI therapy, respectively. The follow-up time in these two patients was 7.0 years and 6.8 years, respectively, and might have been a limitation due to the relatively short time span. Despite the loss of some differentiated characteristics, PDTC retains the ability to produce Tg [16]. The role of Tg in predicting survival and recurrence after initial treatment in PDTC patients is not yet well-established. Former studies showed a reduced recurrence-free survival and DSS in PDTC patients with detectable Tg after the initial surgery and RAI therapy compared to patients with undetectable Tg levels [16,17]. In our cohort, patients with detectable Tg levels (≥0.5 ng/mL) were significantly associated with a worse DSS compared to patients with undetectable Tg in the univariate analysis, which could not be confirmed in the multivariate analysis.

Patients with an incomplete response to the initial therapy at the first diagnostic control and correlating structural disease (S-ED) presented with persistent structural disease until the last follow-up and could not achieve a switch to the NED or B-ED group over time in any case. Most patients developed further distant metastases during the follow-up. The higher rate of distant metastases in PDTC compared to DTC patients is well known [17,18]. While around 25% of patients with PDTC retain their ability to concentrate I-131, the majority of patients become radioiodine refractory resulting in a negative WBS [19,20]. With the loss of RAI avidity, ^18^F-FDG-PET/CT imaging became crucial in these patients. In our study, radioiodine refractoriness could be observed in around half of the study population at the primary presentation and first diagnostic control, and in almost all patients with structural disease at the end of the study. This finding was in line with the study of de la Fouchardière et al. [15], who also reported radioiodine refractory behavior in one third of their PDTC study patients. The ^18^F-FDG-PET/CT scanning confirmed all metastases detected in the I-131 WBS and was able to further reveal I-131-negative lesions. Once distant metastases were detected, mainly by ^18^F-FDG-PET/CT, patients were classified as S-ED and survival was significantly shortened despite multimodal therapy and regardless of the RAI avidity in accordance to Wang et al. [11]. The high rate of distant metastases and their generally strong influence on survival in thyroid cancer patients was already reported by other studies [17]. In the univariate and multivariate analyses, the PET-positivity of distant metastases was the only significant prognostic factor for a shorter DSS, whereas radioiodine avidity had no significant effect on the DSS. Ibrahimovic et al. [17] also reported on the M1 status as an independent predictor of a worse DSS besides the pT4a stage in the uni- and multivariate analyses. Sanders et al. [21,22] recommended considering RAI therapy in PDTC patients due to the potential benefit and relatively low side effects; however, no impact on survival could be found, which was in line with our results.

Age ≥ 55 years and metastatic disease at primary presentation were significantly associated with a worse DSS, which, however, could not be confirmed in the multivariate analysis. Similar to these results, former studies demonstrated that an age older than 45 years and M1 stage in PDTC patients represent an independent predictor of a worse DSS [1,17]. This study monitored the clinical course of patients with PDTC over a mean of 5.1 years. Although this was a relatively long follow-up time in this particular disease, it may represent a limitation. Furthermore, due to the retrospective design of the study and the rarity of the disease resulting in a relatively small cohort, the statistical power of the analysis was limited. Thyroglobulin antibodies were only present in a minority of the patients and, therefore, could not be included in the analysis in a convincing way. This was indeed a limitation of the study, which could not be overcome. However, although the recovery was less sensitive than the direct measurement of the antibodies, we are convinced that an undisturbed recovery adds confidence to the validity of the measured thyroglobulin.

## 5. Conclusions

In conclusion, with response to the initial surgery and RAI therapy, PDTC patients can achieve an excellent outcome and a further follow-up should be refined based on findings at the first diagnostic control similar to DTC. However, particular attention must be given to patients with an incomplete response to initial therapy, especially metastatic patients who become mostly radioiodine refractory and show a significantly shorter survival, which makes accurate staging by ^18^F-FDG-PET/CT imaging (between initial RAI therapy and first diagnostic control) crucial in these patients.

## Figures and Tables

**Table 1:** Patient characteristics.

Age (Years)	57 ± 19
≥55 years	28
Female	26/47
Extrathyroidal extension	9/47
Mean initial RAI dose (GBq)	6.2 ± 1.9
Mean cumulative RAI dose (GBq)	14.2 ± 8.7
Mean RAI therapy cycles (range)	1 (0–3)
Mean stimulated Tg at initial RAI therapy (ng/mL)	1305 ng/mL (<0.05–4109 ng/mL)

RAI, radioiodine; GBq, gigabecquerel; Tg, thyroglobulin.

**Table 2:** TNM classification (7th edition of the American Joint Committee on Cancer).

TNM Classification	*n* = 47
pT1b	3
pT2	6
pT3	34
pT4a	3
pT4b	1
pN0/pNx	33
pN1a	10
pN1b	4
cM0/cMx	29
cM1	18
pR0	28
pR1	9
pR2	1
pRx	9

P, pathological; c, clinical; T, tumor; N, node; M, metastasis.

**Table 3:** Prognostic risk factors for poorer DSS in the uni- and multivariate analyses; DSS, disease-specific survival.

Covariate	Level	Univariate Analysis	*p*-Value	Multivariate Analysis	*p*-Value
		HR (95% CI)		HR (95% CI)	
Age	<55	3.756 (1.027–13.738)	0.045	0.284 (0.030–2.669)	0.271
	≥55 years				
cM stage at primary presentation	M0/Mx	9.445 (2.747–32.479)	0.001	2.692 (0.524–13.815)	0.235
	M1				
Stimulated Tg-level at first control (ng/mL)	<0.5	14.700 (1.872–115.440)	0.011	4.772 (0.313–72.826)	0.261
	≥0.5				
PET-positivity of metastases	negative	14.144 (1.807–110.714)	0.012	10.088 (1.164–87.386)	0.036
	positive				

HR, hazard ratio; CI, confidence interval; R, resection; RAI, radioactive iodine; Tg, thyroglobulin; M, metastases; PET, positron-emission-tomography.

## Data Availability

The data presented in this study are available upon reasonable request from the corresponding author.
